# Intertwined Relationship of Mitochondrial Metabolism, Gut Microbiome and Exercise Potential

**DOI:** 10.3390/ijms23052679

**Published:** 2022-02-28

**Authors:** Saba Imdad, Wonchung Lim, Jin-Hee Kim, Chounghun Kang

**Affiliations:** 1 Molecular Metabolism in Health & Disease, Exercise Physiology Laboratory, Sport Science Research Institute, Inha University, Incheon 22212, Korea; sb.imdad22@gmail.com; 2 Department of Biomedical Laboratory Science, College of Health Science, Cheongju University, Cheongju 28503, Korea; 3 Department of Sports Medicine, College of Health Science, Cheongju University, Cheongju 28503, Korea; wonchlim@gmail.com; 4 Department of Physical Education, College of Education, Inha University, Incheon 22212, Korea

**Keywords:** regular exercise training, gut microbiome, mitochondrial plasticity, short chain fatty acids, metabolism, microbial metabolites

## Abstract

The microbiome has emerged as a key player contributing significantly to the human physiology over the past decades. The potential microbial niche is largely unexplored in the context of exercise enhancing capacity and the related mitochondrial functions. Physical exercise can influence the gut microbiota composition and diversity, whereas a sedentary lifestyle in association with dysbiosis can lead to reduced well-being and diseases. Here, we have elucidated the importance of diverse microbiota, which is associated with an individual’s fitness, and moreover, its connection with the organelle, the mitochondria, which is the hub of energy production, signaling, and cellular homeostasis. Microbial by-products, such as short-chain fatty acids, are produced during regular exercise that can enhance the mitochondrial capacity. Therefore, exercise can be employed as a therapeutic intervention to circumvent or subside various metabolic and mitochondria-related diseases. Alternatively, the microbiome–mitochondria axis can be targeted to enhance exercise performance. This review furthers our understanding about the influence of microbiome on the functional capacity of the mitochondria and exercise performance, and the interplay between them.

## 1. Introduction

The human body is extensively occupied by the microbiome, both externally (skin) and internally (gut and mucosal environments). This ecosystem of microbes has been increasingly researched over the past two decades, facilitated by the development of next-generation sequencing (NGS). Technological advancements in NGS have enabled the culture-independent analysis of the microbiome and its composition [1]. The resultant breadth of our knowledge is expanding, and the gut microbiome has revealed itself as not just a passive bystander, but an active player in multiple host functions, such as metabolism, circadian rhythmicity, nutritional responses, and immunity [2,3].

Exercise is widely accepted as a promising therapeutic strategy for human health, as it modulates specific metabolic signaling pathways [4]. Physical exercise directly benefits skeletal muscle metabolism and systemic energy homeostasis [5]. The adaptation of skeletal muscles to exercise involves muscle contraction via molecular pathways coupled to adenosine 5′-triphosphate (ATP) biosynthesis along with energy expenditure, which is achieved by the activation of various sensors such as mitogen-activated protein kinases (MAPKs), sirtuins (SIRTs), and adenosine monophosphate (AMP)-activated protein kinase (AMPK) [6,7,8]. The exercise-induced stimulus is then transduced to downstream transcription factors of the target genes associated with glucose and lipid metabolism, as well as mitochondrial biogenesis. Mitochondria are vital for the sustenance of life because of their role in ATP production and β-oxidation of fatty acids. In this review, we have highlighted the translated effects of microbes by emphasizing the role of microbiota derived metabolites, their relationship with exercise, and their interconnection with mitochondria and metabolic homeostasis.

## 2. Human Gut Microbiome

The human microbiota represents a complex mini-ecosystem of trillions of microbes which along with its genes is called a ‘microbiome’. They reside at various sites in the human body, such as the urogenital tract, reproductive tract, skin, oral cavity, and gut, which by far consists of the greatest density and diversity of microorganisms [9], consisting of 10^14^ microbial cells [10]. The anaerobic bacteria are the most abundant colonizers of the gut, compared to the other less abundant archaea, fungi, protozoa, and viruses. The bacterial cell numbers (3.9  ×  10^13^) are large enough to almost equate the human cells (3.0 × 10^13^) in the body [9]. However, the microbial gene set is ~150 times larger than the human gene complement [11]. Hence, the large number of microorganisms inhabiting the human body has synonymized ‘microbiome’ with the term ‘second human genome’ [12].

### 2.1. Gut Microbiota and Homeostasis

The gut microbial consortia live in a mutualistic symbiosis with the host and play an essential role in homeostasis and maintaining overall host health, by markedly impacting gut development, regulating immunity, aid digestion and nutrient absorption [13]. Microbiota feed on the undigested food remains, primarily in the colon, to produce vitamins, short-chain fatty acids (SCFAs), and break down toxic substances [14,15,16]. The major contribution of the gut microbiome to host physiology is linked to microbial metabolism [17]. The gut microbiota is responsible for fulfilling approximately 10% of our daily calorie consumption, partly by synthesizing SCFAs from complex non-digestible plant-based polysaccharides in the proximal colon. SCFAs are a subset of fatty acids that can enter the systemic circulation and influence human physiology [18,19]. SCFAs protect the gut epithelium by forming tight junctions to prevent intestinal permeability or a leaky gut. In the case of dysbiosis, the alteration of gut microbiota, which may be represented as the Bacteroidetes to Firmicutes ratio, reduces the levels of junction-forming proteins and results in a leaky gut, leading to the potential translocation of lipopolysaccharides (LPS) or even bacteria to other parts of the body. This event triggers an immune response, in which LPS binds to and activates toll-like receptor-4 (TLR-4), leading to the downstream recruitment of adaptor proteins for the activation of interleukin (IL)-1-associated kinase, tumour necrosis factor receptor-associated factors (TRAFs), c-Jun N-terminal kinases (JNKs), and IκB kinase (IKK). Furthermore, JNK and IKK can induce the phosphorylation of serine residues in insulin receptor substrates (IRSs), an important step in the establishment of insulin resistance. The IKK complex also activates NF-κB for its translocation to the nucleus, initiating an inflammatory response [20].

The gut microbiome is highly malleable, especially in the early years after birth and throughout the lifetime, because the colonization of the gut is influenced by a multitude of intrinsic and extrinsic factors, including diet, host genetics, age, lifestyle, and usage of medicines [21,22,23,24,25]. The gut microbial habitat can be profoundly shaped by the external environment and the host immune system [26,27]. One of the mechanisms by which the gut microbiome controls body composition and lipid absorption is through the circadian transcription factor nuclear factor interleukin 3 (NFIL3). It was revealed that the oscillation amplitude of nfil3 in intestinal epithelial cells (IECs) was regulated by the microbiome in a group 3 innate lymphoid cell-dependent manner as well as via signal transducer and activator of transcription 3 (STAT3), presenting a molecular link between the microbiome, host metabolism, circadian clock, and immune system [28]. The human gut microbiome has shown association with other factors like age. A cross-sectional gut microbiota study of healthy Korean population (n: 890) revealed a greater abundance of *Prevotella* enterotype, which is peculiar to Korean population when compared with other countries. The authors found a strong association between gut microbiota composition and age of the cohort. Additionally, the abundance of Bacteroidia and Clostridia members varied with the dietary routine, body mass index (BMI), and stool frequency of the host [29].

### 2.2. Gut Microbiota-Derived Metabolites Regulate Host Physiology

#### 2.2.1. Short-Chain Fatty Acids (SCFAs)

**Figure 1:**
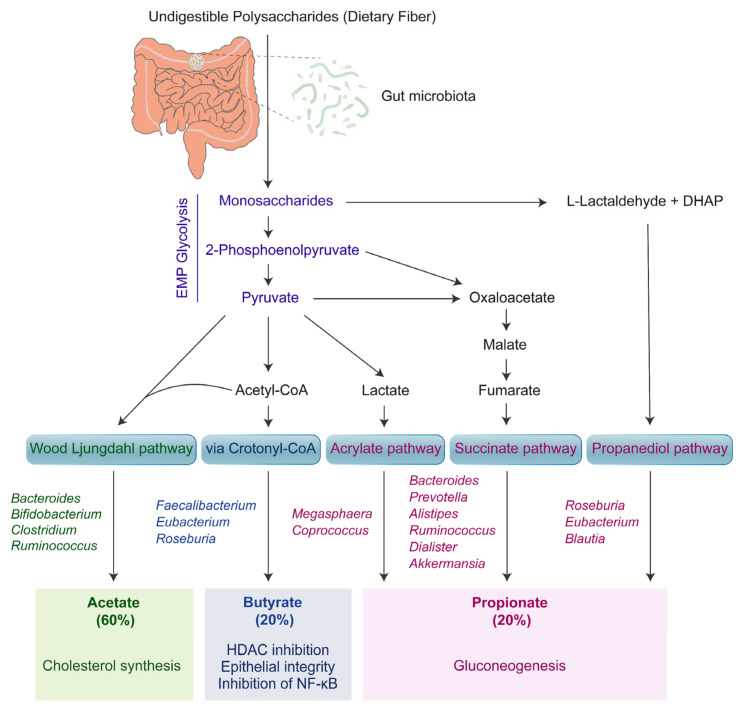
Short-chain fatty acid metabolism by the bacterial gut microbiota and their role in gut homeostasis. EMP stands for Embden–Meyerhof–Parnas pathway. The bacteria involved in the formation of SCFAs via specific pathways are mentioned.

The most abundant and well-studied fecal SCFAs are butyrate, propionate, and acetate (90–95% of the SCFAs), that reach a concentration of 130 mM in the intestinal lumen [30]. SCFAs are microbiome-derived anaerobic fermentation end-products obtained from pyruvate catabolism via different pathways (Figure 1) [31,32,33]. The concentration of SCFAs in the human colon has a relative molar ratio of 60:20:20 for acetate-butyrate-propionate [34]. Acetate and propionate are produced by members of the gram-negative phyla Bacteroidetes and Actinobacteria predominant in the intestinal tract, while butyrate is produced by members of the gram-positive phylum Firmicutes (*Eubacterium rectale*, *Faecalibacterium prausnitzii*, and *Roseburia* spp.) [35]. G-protein-coupled receptors (GPCRs) are the cognate receptors for SCFAs, where GPR43 and GPR41, later renamed as free fatty acid receptors (FFAR) 2 and 3, respectively, are well-studied thus far. Others include GPR109a and GPR164, which are expressed in the gastrointestinal mucosa, immune and nervous systems [36,37]. SCFAs are absorbed by the colonocytes via SCFA-specific GPCRs to preserve the regulatory T-cell (Treg) pool in the rodent mucosa, thus inhibiting intestinal inflammation [38,39]. Moreover, SCFA-mediated nucleotide-binding domain and leucine-rich repeat pyrin 3 domain (NLRP3) inflammasome activation leads to the production of a basal level of IL-18 that regulates the microbial composition and subdues overt inflammatory responses [40].

Despite being less abundant, butyrate is the most favorable energy source and is almost completely utilized by intestinal epithelial cells. Beyond energy provision, the important roles of butyrate include histone deacetylase (HDAC) inhibition and promotion of physiological hypoxia by stabilizing hypoxia-inducible factor (HIF), which serve to target barrier-protective genes and influence mucosal homeostasis [41]. Butyrate generally reduces gut mucosal inflammation by favoring the production of anti-inflammatory cytokines, such as transforming growth factor β (TGF-β), IL-10 secretion from T-cells, and IL-18 secretion in the intestinal epithelium, and inhibiting the activity of pro-inflammatory signaling molecules, such as nuclear factor kappa B (NF-κB). Anti-inflammatory and immune suppressive Tregs are also modulated by the action of butyrate [31,41,42,43,44]. Besides, the members of microbiota from order Clostridiales are known to provide TGF-β enriched environment for promotion and accumulation of Tregs in the gut. A study reported that butyrate acts through SP1 mediated HDAC inhibition in IECs to regulate the expression of TGF-β1. TGF-β1 was modulated by the supernatants of members of Firmicutes and Fusobacteria, in HT-29 cells [45]. Moreover, butyrate can activate colonocyte peroxisome proliferator-activated receptor γ (PPARγ) signaling, which induces butyrate β-oxidation by consuming oxygen and thereby regulating oxygen concentration in the lumen of gut [46]. Therefore, butyrate dynamically regulates intestinal homeostasis by driving colonocyte hypoxia. The action of antibiotics can suppress PPARγ signaling and reduce butyrate levels in the colon, which in turn minimizes Tregs [47,48], and exacerbates inflammation of the colonic mucosa. Invading pathogens take advantage of the escalating inflammation, which shifts the metabolism of colonocytes towards anaerobic glycolysis, disrupting epithelial hypoxia. Hypoxia maintains the F-actin filament disorganization and occludin mis-localization which impairs the endocytotic process utilized by several invading pathogens, such as, *Shigella* and *Yersinia* [49,50,51,52]. In addition, butyrate enhances insulin sensitivity, decreases intestinal cholesterol synthesis, and suppress colitis and the risk of colorectal cancer [41,44,47,53,54]. It has been shown that peak oxygen uptake, an indicator of cardiovascular fitness, is correlated with gut microbial diversity, increased fecal butyrate production, and increased abundance of butyrate-producing taxa (Clostridiales, *Roseburia*, *Lachnospiraceae*, and *Erysipelotrichaceae*) in physically fit participants, independent of diet [53].

Propionate can also activate PPARγ signaling [54]. In addition, propionate and acetate can suppress TLR-4 mediated pro-inflammatory cytokine responses. Unlike butyrate, propionate and acetate are consumed by the host intestine to a much lesser extent and are transported systemically to other organs, such as the adipose tissue and liver. Excess unmetabolized SCFAs are utilized as precursors of gluconeogenesis (propionate) and lipogenesis (acetate and butyrate) (Figure 1), particularly by hepatocytes [47]. Higher SCFA excretion in feces has been linked with gut dysbiosis, gut permeability, excessive adiposity, and cardiometabolic dysregulations, such as obesity and hypertension [55].

#### 2.2.2. Hydrogen Sulfide

Hydrogen sulfide (H_2_S) also acts as a messenger, similar to SCFAs, for modulating gene expression, epigenetic modifications, and metabolism in intestinal epithelial and immune cells. The source of pleiotropic H_2_S is either host colonic epithelial cells or intestinal microbiota, where cysteine degradation by gut microbiota has been recognized as a predominant pathway for microbial H_2_S production [56]. Generally, it is produced by the degradation of sulfur amino acids, by colonic sulfate reducing bacteria such as *Desulfovibrio piger* and *D. desulfuricans*, *Desulfobacter, Desulfobulbus,* and *Desulfotomaculum* [57] (Table 1). H_2_S may be beneficial for colonocyte mitochondrial respiration at low concentrations via the sulfide quinone reductase-associated metabolism of sulfide [58]. However, colonic H_2_S levels can directly regulate oxidative phosphorylation (OXPHOS) in IECs, where increased H_2_S levels can inhibit the activity of one of the major complexes of electron transport chain (ETC), complex IV, or cytochrome c oxidase (COX) [59], leading to detrimental effects. Human colonic epithelial cells with dysfunctional mitochondria are more prone to barrier function defects. Moreover, H_2_S (at 2 mM) was found to inhibit acyl-CoA dehydrogenase enzyme responsible for butyrate oxidation, leading to the impairment and disruption of gut homeostasis [60]. Moreover, H_2_S induces DNA damage in epithelial cells, inhibits SCFA metabolism, and compromises the mucus barrier by inducing breaks and permitting the exposure of luminal contents to the underlying tissues [61].

#### 2.2.3. Trimethylamine-N-oxide (TMAO)

TMAO is produced through meta-organismal steps from precursors like betaine and its metabolite γ-butyrobetaine and choline, which are converted to trimethylamine (TMA) by the gut microbiota belonging to Firmicutes and Proteobacteria phyla, in the intestine [62]. TMA is absorbed through the intestinal wall and transported to the liver for subsequent oxidation into TMAO, by the action of flavin-containing monooxygenases (FMO 1 and 3) [63,64]. These dietary precursors are abundant in red meat, eggs, dairy products, and fish. The nutrients like L-carnitine, phosphatidylcholine, and choline are especially abundant in high-fat foods, which can increase the risk of atherosclerosis [65,66]. Additionally, TMAO can be directly absorbed through the gut after consumption of TMAO-rich foods like shellfish and fish. Human studies have shown an elevated level of plasma TMAO in patients with obesity [67], diabetes [68], or risk of diabetes [69]. Moreover, an increased TMAO concentration is also reported in the plasma of colorectal cancer (CRC) patients, where putative cell cycle progression and Wnt signaling pathways have been identified, that are shared by both TMAO and CRC [70]. The potential mechanism behind TMAO-induced cancerous phenotype is by increasing the expression of pro-inflammatory IL-6 gene and chemokine ligands, which also play a part in cancer progression, as well as tumor necrosis factor alpha (TNFα), as reported in certain infections and metabolic diseases, like diabetes and chronic kidney disease [71]. Similarly, an association between plasma TMAO and several TMA-producing fecal bacteria was reported, pointing towards a possible contribution to inflammatory and cardiometabolic risk pathways in multiethnic adiposity phenotype study [72]. The key TMA-producing players in human fecal samples belonged to the three major bacterial taxa; Actinobacteria, Firmicutes, and Proteobacteria, but the majority were associated to the members of *Clostridium* XIVa and a specific *Eubacterium* [73].

#### 2.2.4. Succinate

Succinate is produced by both the gut microbes and the host. In human gut, succinate is produced by anaerobic fermentation of oligo/poly-saccharides, ranging in concentration from 1 to 3 mM in intestine and feces [74]. Microbiota derived succinate tends to accumulate to a lesser extent in the gut because of its intermediate nature in propionate synthesis pathway (Figure 1) [34]. Members of Bacteroidetes phylum are major producers of succinate in the gut [75], where they modulate intestinal inflammation by activating immune cells, such as dendritic cells [76,77] and activate proinflammatory programs [78]. While a number of anaerobic bacteria produce succinate naturally, facultative anaerobes like *E. coli* and *Corynebacterium glutamicum* are capable of producing succinate from carbohydrates via reductive tricarboxylic acid (TCA) cycle, under oxygen deprived conditions [79,80]. Succinate is also generated by the host, in the TCA cycle as a product of 2-oxoglutarate-dependent dioxygenases enzyme reaction, and its accumulation can inhibit histone and DNA demethylation, which can alter the epigenetic status of the cell to influence gene expression. Under normoxic conditions, succinate can inhibit prolyl-hydroxylases (PHDs), which are critical for regulating the master transcription factor of O_2_ homeostasis, HIF-1α. In normoxia, HIF-1α proline residues are hydroxylated by PHDs, which is a signal for the poly-ubiquitination of HIF-1α by VHL (von Hippel-Lindau tumor suppressor) protein and subsequent degradation by proteasome. In contrast, under hypoxic conditions PHD’s function is impaired, leading to the accumulation and translocation of HIF-1α or HIF-2α to the nucleus where it regulates gene expression related to metabolism, erythropoiesis, immune and stem cell function [81]. Apart from HIF stabilization, epigenetic regulation, and inflammation, the role of cytosolic succinate has been implicated in reactive oxygen species (ROS) production [82]. Additionally, some evidence exists regarding the role of intracellular succinate in thermogenesis [83] and gluconeogenesis [84]. On the extracellular level, succinate interacts with its cognate GPCR receptor, succinate receptor 1 (SUCNR1). As mentioned above, succinate has long been known as an inflammation boosting signaling molecule. Conversely, its anti-inflammatory effects had been ascribed to SUCNR1 [85]. In human obesity, the pivotal factor for the active resolution of acute inflammation by macrophages is SUCNR1 [86]. Moreover, succinate has been reported to behave as a pH sensing metabolite, acting in a paracrine fashion to remodel skeletal muscles during exercise via SUCNR1 signaling pathway, to coordinate skeletal muscle adaptation [87,88]. In the light of such evidence, succinate cannot be conclusively tagged as beneficial or harmful.

#### 2.2.5. Secondary Bile Acids

Primary bile acids (BAs) are produced in liver from cholesterol by the action of the rate limiting enzyme cholesterol-7α hydroxylase. Primary BAs cholate (CA) and chenodeoxycholate (CDCA) are conjugated with either taurine or glycine and stored in the gall bladder, until being released into the small intestine after a meal [89], where around 95% of the acid is absorbed to be transported back to the hepatocytes [90]. The unabsorbed primary BAs enter the colon, where they are modified by the gut microbiota for conversion into secondary BAs. For decades, microbes have been modifying human BAs by distinct mechanisms, such as 7α-dehydroxylation, dehydrogenation, epimerization of the cholesterol core, by deconjugating glycine or taurine, and by a recently revealed mechanism of conjugating amino acids to BAs. The altered chemistry of secondary BAs have health implications and they have been associated with diseases like cancer, inflammatory bowel disease (IBD) and liver cirrhosis [91]. Primary and secondary BAs have endocrine functions [92]. Gut microbiota metabolise 5–10% of the BAs, modulating the associated signaling pathways via nuclear farnesoid X receptor (FXR) and the G-coupled membrane protein 5 (TGR5) [93,94]. In contrast, BAs can modulate gut microbial composition by activating genes of the innate immune system in the small intestine, both directly as well as indirectly [92]. Members of *Clostridium*, clusters XIVa and XI and *Eubacterium* genera, belonging to Firmicutes phylum are capable of producing secondary BAs [95]. Deoxycholic acid (DCA) is a secondary BA and CA derivative, and its elevated levels are linked with obesity and cancer in mice, emphasizing the importance of BA bioconversion for host metabolism [96]. In addition, DCA and CA also act as antimicrobial agents [97]. Further details concerning microbial metabolites are given in Table 1.

**Table 1:** Gut microbiota-derived metabolites and their role in disease.

Gut Microbiota-Derived Metabolites	Metabolite Producing Bacteria	Metabolite Producing Bacterial Proteins/Genes	Role in Metabolic Disorders and/or Mitochondrial Diseases
H_2_S	Cysteine catabolic bacteria: *Fusobacterium, Clostridium, Escherichia, Salmonella, Klebsiella, Streptococcus, Desulfovibrio,* and *Enterobacter* [98]	Sulfate reduction operon (*dsrAB*), cysteine desulfhydrase (*dcyD*), *yhaOM* operon, methionine gamma-lyase (*mgl*), cysteine aminotransferase + 3-mercaptopyruvate sulfurtransferase (*aspC* + *sseA*), cystathionine beta-lyase (*metC*), cystathionine beta-lyase like; repressor of maltose regulon (*malY*), cysteine synthase A (*cysK*), cysteine synthase B (*cysM*), cystathionine gamma-lyase (*mccB*) [99]	Mutation in parkin encoding gene *park2*, is associated with autosomal recessive Parkinson’s disease [100]. The E3 ubiquitin ligase activity of parkin is amplified upon protective sulfhydration, which facilitates mitophagy. Ubiquitin specific peptidase 8 (USP8) is required for the recruitment of parkin to dysfunctional mitochondria. USP8 was also reported to be post-translationally modified by sulfhydration in response to H_2_S treatment, easing its interaction with parkin and facilitating mitochondrial docking in a cardiomyopathy db/db mouse model [101,102].
TMAO	*Anaerococcus hydrogenalis, Clostridium asparagiforme, Clostridium hathewayi, Clostridium sporogenes, Escherichia fergusonii, Proteus penneri, Providencia rettgeri,* and *Edwardsiella tarda* [62]	choline-TMA lyase (*cutC*), carnitine oxygenase (*cntA*), betaine reductase (*grdH*) [103]	Cell culture studies showed that TMAO promotes chronic kidney disease (CKD) by activation of NF-κB. Non-lethal inhibitor of gut microbial TMA production attenuated CKD and cardiac hypertrophy, in adenine induced CKD mouse model [104]. Increased TMAO concentration in cardiac mitochondria impairs pyruvate and fatty acid oxidation [105]. The same group, later reported a protective role of elevated TMAO, by restoration of fatty acid oxidation and cardiac functionality in the experimental model of right ventricular heart failure [106].
Succinate	*Prevotella copri* [84], *Propionibacterium* spp., *Bacteroides fragilis, Faecalibacterium prausnitzii, Citrobacter freundii, Ruminococcus* spp., *Akkermansia muciniphila, Bacteroides fragilis, Parabacteroides distasonis, Blautia wexlerae, Succinivibrio dextrinosolvens, Alistipes indistinctus, Paraprevotella* spp. [85], *Bacteroides thetaiotaomicron* [107], *Anaerobiospirillum succiniciproducens*, *Actinobacillus succinogenes*, *Mannheimia succiniciproducens* [108]	6-phosphogluconolactonase (*pgl*), transketolase (*tktA*), transaldolase (*talB*) glucose-6-phosphate dehydrogenase (*zwf*), 6-phosphogluconate dehydrogenase (*gnd*) [109,110]	LPS stimulated macrophages shift from OXPHOS to glycolysis and increase succinate levels by oxidation via mitochondrial succinate dehydrogenase (SDH), along with a higher mitochondrial membrane potential, to drive mitochondrial ROS production. Succinate via SDH has a role to play in the repurposing of mitochondrial metabolism to promote pro-inflammatory macrophages [111].
Secondary bile acids	*Bacteroides fragilis, Bacteroides vulgatus, Clostridium perfringens, Eubacterium, Lactobacillus* and *Bifidobacterium* [112]	Bile salt hydrolase (*bsh*) gene encodes the deconjugation of conjugated primary BAs, bile acid-inducible (*bai*) gene cluster for the 7α/β-dehydroxylation pathway [113]	The secondary bile acid lithocholic acid (LCA) was found to be reduced in the fecal samples of nonalcoholic fatty liver disease (NAFLD) children, when assessed by tandem mass spectrometry (UPLC-MS/MS). *Eubacterium* and *Ruminococcaceae* bacteria were significantly and positively correlated, whereas, the abundance of pathogenic *Escherichia coli* was negatively associated with fecal LCA levels, in NAFLD children [114].
Tryptophan catabolites	*Bacteroides ovatus* and other spp. produce indole, *Clostridium sporogenes* and other spp. produce indolepropionic acid (IPA), *Bifidobacterium adolescentis* and other spp. produce indolelactic acid (ILA), *Lactobacillus acidophilus* and other spp., *Anaerostipes hadrus, Anaerostipes caccae, Peptostreptococcus russellii, Peptostreptococcus anaerobius, Peptostreptococcus stomatis, Butyrivibrio fibrisolvens, Ruminococcus gnavus* [115]	Phenyllactate dehydratase gene cluster (*fldAIBC*) is involved in indoleacrylic acid (IA) and IPA formation [116], tryptophanase (*tnaA*) is involved in indole formation [117], aromatic-amino-acid aminotransferase (*tyrB*) and indolelactate dehydrogenase (*fldH*) are involved in the formation of indolealdehyde (IAld) and ILA [115], Tryptophan decarboxylase (RUMGNA_01526) converts tryptophan into tryptamine [118].	IA was suggested to have a potentially protective role in lipid metabolism, where it is reduced in mice on a high-fat diet (HFD) vs low-fat diet and is involved in diminishing hepatocyte lipogenesis via cytokines [119]. Gut microbiota derived-metabolite IPA was identified as modulator of mitochondrial respiration in cardiomyocytes and affected cardiac function, in an isolated perfused heart model [120].

## 3. Crosstalk between Mitochondria and the Gut Microbiome

Mitochondria plays a significant part in energy metabolism, ion homeostasis, intracellular redox status, cytosolic calcium regulation, and cell growth [86]. In order to fulfill these functions, mitochondria communicates with the cell via several mechanisms, such as the induction of the apoptotic pathway by the release of cytochrome c, AMPK mediated regulation of mitochondrial fission and fusion, production of ROS to regulate gene expression, and immune response activation by releasing mitochondrial DNA [81]. However, the complexity of mitochondrial regulation is increased when gut microbiome comes into play as a modulator. Both the mitochondria and the microbiome are maternally inherited. Moreover, mitochondria, amongst other organelles, have been reported to be the most responsive organelle to microbiota signaling [121]. This section describes the dynamic interplay of mitochondria and gut microbiota and its effect on the cellular phenotype and overall physiology by understanding the mechanistic features of their crosstalk.

### 3.1. Mitochondrial Bioenergetics and Gut Microbiota Synergy

Mitochondrial (mt) DNA is located within the mitochondrial matrix, in the ‘nucleoids’ region and has a molecular mass of 10^4^ kDa [122]. Phylogenetic analysis of mitochondrial genome showed that mitochondria have descended from the primitive α-Proteobacteria, which represents the first known case of ETC-mediated ATP synthesis [81]. The circular double stranded mt-DNA consists of a guanine-rich heavy strand and a cytosine-rich light strand, while most of the mt-DNA components are coded by the former strand [82]. The human mt-DNA is 16.569 kb in size and possesses 37 genes, encoding two rRNAs and 22 tRNAs. Additionally, it generates polycistronic transcripts for the expression of 13 mitochondrial proteins (~1% of the mitochondrial proteome), which contributes to the functional components of four ETC complexes out of five, which makes it crucial for ATP synthesis [123,124]. Unlike nuclear DNA, mt-DNA lacks histones, and most of the genes also lack introns. This contributes to the susceptibility of mt-DNA to oxidative stress [125]. However, short introns are part of the non-coding hypervariable D-loop region (1.1 kb) of the mitochondria, which possesses the start site for mt-DNA replication and promoters for mt-gene transcription [126]. D-loop fragments vary between individuals and have applications in population genetics and forensic medicine [127].

Mitochondrial DNA copy number can be associated with ATP production and mitochondrial enzyme activity and is regulated in a tissue specific manner. Different cell types have different physiological requirements. For example, tissues, such as the heart, brain, skeletal muscle, and brown fat, have higher OXPHOS activity, which is associated with mitochondrial fusion that elongates mitochondria and is mainly regulated by mt-DNA [128]. Mitochondrial DNA copy number can also influence the methylation status of nuclear DNA at specific loci, to alter the gene expression profile that could impact human health and disease via perturbed cell signaling [129].

Metabolic pathways, such as glycolysis and mitochondrial respiration, are the major sources of ATP. The conversion of glucose to pyruvate via glycolysis, initiates the mitochondrial function of energy production by OXPHOS. The 3-carbon pyruvate molecule is oxidized to form acetyl coenzyme A (acetyl-CoA; 2-carbon), which is fed into the TCA cycle in the mitochondrial matrix, where it is converted into CO_2_, NADH and FADH_2_. Acetyl-CoA is also derived from β-oxidation of fatty acids for consumption in the TCA cycle. Hence, the TCA cycle is important for the generation of electron carriers for OXPHOS, in the form of NAD(P)H and FADH_2_, which pass electrons through the ETC complexes, in the inner mitochondrial membrane (IMM) and in combination with chemiosmosis, results in the production of ATP, which is transported to the cytosol by adenine nucleotide translocase in exchange for ADP [130]. The gut microbiome is also engaged in food digestion and xenobiotic metabolism through its metabolic pathways and enzymes and is responsible for generating a large proportion of bioactive molecules, such as SCFAs, vitamins, and amino acids. Gut microbiota-derived metabolites are necessary for the interlinked pathways of glycolysis, TCA cycle, OXPHOS, as well as amino acid and fatty acid metabolism.

Mitochondrial gene polymorphism is another phenomenon that can regulate the gut microbiota as demonstrated in a mice study, where the mitochondrial ATP8 synthase gene was mutated at a single location and backcrossed to generate conplastic mice strains. The strain carrying mutated mt-ATP8 synthase resulted in an increased Firmicutes-to-Bacteroidetes ratio, as well as a phenotype correlating with metabolic and inflammatory impairment. According to the report, the mt-ATP8 mutation causes modulation of OXPHOS and glycolysis pathways in a way that shifts the gut microbial composition, as a result of host metabolism. In addition, the results suggest that gut microbiome can act synergistically to regulate mitochondrial bioenergetics of host and thereby can regulate host genomics, which can potentiate the risk of diseases [131]. The influence of mitochondria on the gut microbiome can be read here [132].

Gut microbiome interacts with the host and other tissues via its metabolites and therefore a huge number of microbiota-derived metabolites are part of human metabolome [133]. These active metabolites (vitamin A and B) are produced by the gut microbiota to regulate the activity of chromatin modulating enzymes in the host and are epigenetic modifiers. Histone modifications, such as acetylation and deacetylation, are carried out by acetyl group provided by acetyl-CoA. Being members of the fatty acid family, SCFA metabolites, such as acetate and butyrate, are main substrates for de novo lipid metabolism in rat colonic epithelial cells, that convert SCFAs into acetyl-CoA [134]. Hence, alteration of the gut microbiota is reflected in the epigenome of the cells as well as in the lipid metabolism regulation. These processes highlight the metabolic implications of central mitochondrial functions and their coordination with the gut microbiome, which can profoundly impact physiological processes.

### 3.2. Mitochondrial Plasticity and Gut Microbiota Interaction

Mitochondrial processes require a balance of mitochondrial biogenesis, fission, and fusion and an evolutionarily conserved process of autophagy or mitophagy. Mitochondrial biogenesis involves two transcription factors; mitochondrial transcription factor A (TFAM) and the master regulator, peroxisome proliferator-activated receptor gamma coactivator 1-alpha (PGC-1α) [135]. Mitochondria are the principal sites of OXPHOS, modulated by PGC-1α, and their health is equivalent to metabolic health [136,137]. Mitochondrial morphology is altered to modulate mitochondrial bioenergetics, which involves a balance between fusion and fission regulated by large guanosine triphosphatases (GTPases) of the dynamin family. In mammals, mitochondrial fusion is carried out by mitofusin-1 (MFN-1) and MFN-2, which regulate outer mitochondrial membrane fusion, and optic atrophy protein-1 (OPA-1), which mediates inner mitochondrial membrane fusion. However, GTPase dynamin-related protein-1 (Drp-1) regulates mitochondrial fission along with its mitochondrial anchors: mitochondrial fission protein 1 (Fis1) and mitochondrial fission factor (Mff) [138,139]. Mitochondrial fusion greatly enhances the capacity of ATP synthesis to cope with energy demands during nutrient deprivation. Mitochondria are elongated by inhibiting the fission process via protein kinase A (PKA)-mediated Drp-1 phosphorylation or by MFN-1 deacetylation-induced mitochondrial fusion. Mitochondrial fusion helps cells escape massive autophagy and rescues them from detrimental conditions; thus, it maintains cell viability [140,141]. However, excess nutrients lead to mitochondrial cleavage, which results in the uncoupling of the electron transport chain and decreased ATP production, which is Drp-1-dependent during glucose overload. Moreover, mitochondrial fragmentation is also enhanced by a high-fat diet due to decreased levels of MFN-2 [142,143,144,145]. Mitochondrial dynamics mark the metabolic status of cells and can be considerably influenced by the environmental signals.

The pristine mitochondrial population can be achieved by selectively removing dysfunctional or damaged mitochondria, via mitophagy, which is mediated by two major pathways; damage-induced mitophagy and development-induced mitophagy [146]. Mitochondria subjected to defective depolarized fission are tagged for autophagosome engulfment and subsequent degradation by lysosomes via damage-induced mitophagy, which is driven by PTEN-induced putative kinase (PINK) and E3 ubiquitin-protein ligase (Parkin) [147,148]. Developmental process-induced mitophagy is driven by proapoptotic proteins, such as the adenovirus E1B 19 kDa-interacting protein 3 (Bnip-3) (Bcl-2 family protein) and Nix (Nip3-like protein X/Bnip3L), which translocate mitochondria to autophagosomes by directly interacting with a ubiquitin-like modifier, microtubule-associated protein 1A/1B-light chain 3 (LC3), which in turn is required for autophagosome growth [149,150]. Damaged ROS-generating mitochondria can accumulate due to depletion of LC3 and Beclin 1, a marker of autophagosome formation, leading to the translocation of mitochondrial DNA in the cytosol [151]. Moreover, suppression of mitophagy can disrupt energy metabolism, escalate ROS production, activate the NLRP3 inflammasome, and therefore initiate apoptotic cell death mechanisms [133]. With senescence, mitochondrial biogenesis is reduced and shifts more towards fission than fusion in most tissues, thereby facilitating the occurrence of mitophagy as a protective mechanism [152,153,154]. These mechanisms should remain in an equilibrium state to stabilize the population of healthy mitochondria, since mitochondrial dysfunction, apart from other consequences, can lead to the inability of intestinal epithelial cells to tolerate commensal bacteria [155]. More importantly, the alteration in the cytokine environment such as, an increased expression of TNFα and interferon gamma (IFNγ) during clearance of bacterial infection can disrupt the mitochondrial function, which is counteracted by the concomitant increase in IL-4 and IL-16, for shielding mitochondrial function [156]. Therefore, mitochondrial stress of epithelial cells can mask the benefits of commensal bacteria and they are considered pro-inflammatory threats by the host.

Many studies have focused on the intricate signaling between the gut microbiome and mitochondria, in a variety of perspectives. The microbiome-derived metabolites provide the functional readouts for understanding the different roles of microbiota in health and disease. Microbiome-derived SCFAs are known to directly activate the master sensor of energy stress AMPK, by increasing the AMP/ATP ratio [157]. AMPK regulates metabolism by shutting down ATP consumption and stimulate catabolism induced ATP production [158]. Chronic activation of muscle AMPK can activate nuclear respiratory factor-1 (NRF-1) and increase mitochondrial biogenesis, by increasing cytochrome c and density of muscle mitochondria [159]. AMPK senses lower levels of energy in the cell and as a result can trigger mitophagy through activation of Unc-51-like-kinase 1 (ULK1) and inhibition of mTOR complex 1 (mTORC1) pathway. ULK1 is a serine/threonine kinase mainly found in the endoplasmic reticulum, mitochondria, lysosomes, and cytosol. Upon activation, ULK1 triggers the complex initial phases of autophagosome formation, by phosphorylating autophagy-related protein 13 (ATG13) and focal adhesion kinase family interacting protein of 200-kDa (FIP200) [160]. mTORC1 senses ATP and amino acids to regulate nutrient availability and cell growth, and its activation inhibits autophagy [161]. Additionally, AMPK can promote autophagy by facilitating mitochondrial fission [162]. Moreover, gut microbiota derived SCFAs can influence NADPH oxidase activity through AMPK signaling in a 2K1C rat model of hypertension that had disrupted gut microbiota, decreased number of SCFA-producing bacteria, and increased abundance of *Prevotella*. AMPK/NADPH oxidase signaling pathway is important for regulating inflammatory and oxidative stress responses [163]. SCFAs can also regulate PGC-1α, which may induce mitochondrial biogenesis [164] and regulate glucose metabolism as well as fatty acid oxidation (FAO) [165]. Moreover, AMPK signaling can also lead to the increased expression of PGC-1α in adipose tissue and skeletal muscles [166,167].

### 3.3. ROS and Gut Microbiota Shape Each Other

Excess ROS was historically known to non-specifically oxidize macromolecules and contribute to the intracellular oxidative damage, leading to subsequent cellular dysfunction and ultimately cell death [168]. The long-known role of physiological ROS as a notorious molecule was supplanted by the idea that mitochondrial stress induced-ROS can activate cytosolic signaling pathways, by functioning as a secondary messenger, at sublethal concentrations, to induce cytoprotective effects [169,170]. Mitohormesis is the term used to define the fine balance of ROS level for enhanced mitochondrial performance [169]. The imbalance of ROS production and scavenging which is carried out by enzymatic and non-enzymatic mechanisms and the excessive accumulation of ROS possibly by inefficient mitophagy, can hamper the critical functioning of mitochondria. Additionally, the downstream signaling pathways are altered, leading to a variety of diseases including cancer, where ROS mediates redox signaling to support disease progression [171,172]. Moreover, mt-ROS play part in survival of certain cells by development of their niche and function. It has been demonstrated that mt-ROS produced during OXPHOS in Lgr5+ crypt base columnar cells (CBCs) stimulates the activation of p38 MAP kinase, which regulates Lgr5+ CBC self-renewal and differentiation [173]. Gut commensals are essential to counteract the pathogenic bacteria, augment the immune system [174] and regulate the important gut epithelial barrier function. Bacteria in the gut lumen release small formylated peptides that bind to a distinct class of pattern recognition receptors in the gut epithelium, and activate superoxide anion by NADPH oxidase 1 (NOX1), resulting in increased cellular ROS, other than the mitochondria source [175]. These activities induce the regulatory redox sensor proteins, which are necessary to maintain the gut epithelial barrier function and induce anti-inflammatory IL-10 secretion [59]. Commensal bacteria induced ROS production in epithelial cells can also promote wound healing and the recovery of damaged cells [176]. It has been reported that the mitochondrial genotype can affect mt-ROS production, and thus, the gut microbiota diversity, illustrating that the mechanisms underlying the altered microbiome in connection with the disease phenotype may involve changes in mitochondrial functional capacity [177]. Recently, it has been reported that mt-ROS and other similar mitochondria-related species undergo TLR-dependent signaling, thereby triggering mitohormesis as a negative feedback mechanism to prevent inflammation via tolerance [178]. On the other hand, gut microbiota modulates mt-ROS production by a proinflammatory reaction of succinate oxidization, leading to production of IL-1β [111]. The stimulation of OXPHOS and mt-ROS causes elevation of forkhead box protein P3 (FOXP3) expression, which is achieved by enhanced histone acetylation of Foxp3 promoter, leading to Treg cell differentiation [179]. In addition, T cells respond to infection by generating NOX-2 mediated ROS production, and acetate can re-establish the redox balance in T-cells, possibly through upregulation of HDAC [180]. These studies indicate that the concentration of ROS in the cell is an important determinant of the immune response.

## 4. Influence of Exercise on the Microbiome and Mitochondria

The mechanistic details of the protective effects of moderate-intensity exercise are not known; however, it is well established that moderate-intensity exercise can modulate gut microbiota and induce anti-inflammatory effects in the gut. High-fat diet (HFD)-induced obesity-related adverse effects can be counteracted by high-intensity interval training (six weeks) by increasing the aerobic capacity, alpha diversity, and Bacteroidetes to Firmicutes ratio in mice, more prominently in the distal gut, compared to the chow-fed mice. Manor et al. described a non-monotonic relationship between Bacteroidetes and microbial diversity, suggesting that a single factor such as the Bacteroidetes to Firmicutes ratio cannot define the overall health status with regard to microbiota diversity and composition. Instead, there may be a spectrum of differential sets of beneficial bacteria in each individual to be tagged as a healthy microbiome [181].

Acute and/or chronic exercise can trigger the dynamic processes of mitochondrial biogenesis, fusion, fission, and mitophagy [182]. A study reported that exercise training can increase the metabolic and genetic capacity in relation to the TCA cycle, as opposed to the decreased metabolic capacity in the fecal microbiota of non-exercised mice with HFD-induced obesity [183]. The alpha diversity in the gut microbiome has been associated with eubiosis, i.e., a microbial balance within the body, whereas reduced alpha diversity is linked to several acute and chronic disease conditions [184].

**Figure 2:**
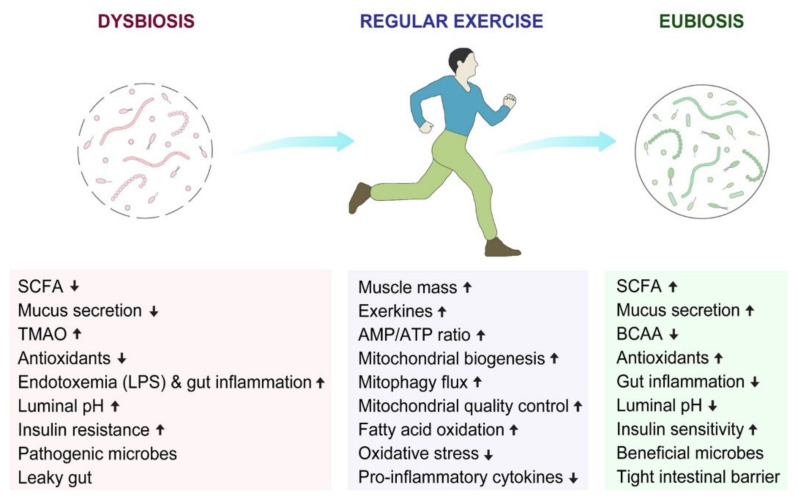
Influence of regular exercise on the gut microbiota in the context of transforming the diseased condition (dysbiosis) to a healthier condition (eubiosis).

It has been demonstrated that voluntary exercise curbs TMAO elevation and decreases myocardial inflammation and fibrosis, leading to the prevention of cardiac dysfunction in western diet-induced obesity [185]. On the other hand, branched-chain amino acids (BCAAs), which comprise three of the nine essential amino acids; leucine, isoleucine, and valine, are obtained from the diet. Heavy-intensity exercise has been reported to reduce the plasma BCAA levels, which are important for individuals showing a high risk of cardiometabolic dysfunction [186]. *Akkermansia muciniphila* has been associated with a healthy intestinal microbiota and in known to increase in abundance in response to physical activity. This bacterium feeds on mucin structures and has a butyrogenic effect in the intestine which strengthens the epithelial barrier integrity [187]. These mucin modulating bacteria also include Lactic acid bacteria, such as *Lactobacilli* and *Bifidobacteria*, which can enhance the production of mucin in human IECs and cause hinderance for the invasion of enteropathogenic *E. coli* [188]. Therefore, exercise can indirectly enhance mucus production by the action of good bacteria, which can increase mucin synthesis via SCFA production [189]. Moreover, SCFAs, in response to aerobic exercise, can decrease the luminal pH (colon) by decreasing the conversion of primary bile acids to secondary bile acids and promoting colonic acidification. The environment created as a result of these changes is more favorable for the growth of healthy commensal bacteria [190,191]. Figure 2 summarizes the beneficial effects of exercise on the dysbiotic gut.

A six-week moderate to intense aerobic exercise (30–60 min) program can induce a shift in the SCFA-producing *Faecalibacterium* and *Lachnospira* spp. and genetic machinery (BCoATe) substantially in lean (*n* = 18, 25.1 ± 6.52 years) versus obese (*n* = 14, 31.14 ± 8.57 years) participants. However, the change in the microbiota composition was reversed in a BMI dependent manner after a sedentary lifestyle (for six weeks) was resumed [192]. The high SCFA concentration due to exercise intervention suggests that the skeletal muscles are being fueled during prolonged contractions. Likewise, in C57BL/6J germ-free mice devoid of microbiota, SCFA supplementation for four weeks can reinforce muscle strength to levels similar to those in mice with gut microbiota [193]. The role of specific bacterial species may be important for enhancing exercise capacity through SCFA and other metabolite production. The genus *Veillonella* was positively correlated with extensive physical exercise in the post-race samples of marathon runners. They found that *Veillonella* inoculation into mice can significantly increase the exhaustive treadmill run time. Lactate is the sole carbon source for *Veillonella.* Therefore, a shotgun metagenomic analysis was performed in a cohort of elite athletes, where major pathway genes involved in the conversion of lactate to propionate were found at a higher relative abundance postexercise. *V. atypica* improves run time via its metabolic conversion of exercise-induced lactate into propionate, providing evidence that exercise can independently and consistently switch the structure and composition of the gut microbiome [181,194]. Additional evidence came from the fecal metagenomic shotgun sequencing and metabolomics (fecal and urine) analysis of international athletes from 16 different sports. They reported no difference in microbial diversity based on sports type. Altogether, the analysis was dominated by a single or a combination of five species, namely *Eubacterium rectale, Polynucleobacter necessarius, Faecalibacterium prausnitzii, Bacteroides vulgatus,* and *Gordonibacter massiliensis.* Species such as *Bifidobacterium animalis, Lactobacillus acidophilus, Prevotella intermedia,* and *F. prausnitzii* were associated with athletes participating in high dynamic component sports. Such studies highlight the type and load of exercise training as a contributing factor in the observed gut microbiome differences, in the absence of dietary changes [195]. Similarly, another study showed no difference in gut microbiota alpha and beta diversity among athlete types. However, there was significant correlation with the relative abundance of gut microbiota at the genus and species level with athlete types; *Faecalibacterium, Sutterella, Clostridium, Haemophilus,* and *Eisenbergiella* were the most abundant in bodybuilders, while *Bifidobacterium* and *Parasutterella* were the least abundant. Additionally, resistant or aerobic exercise training was associated with dietary patterns with regard to gut microbiota diversity [196]. Exercise induced gut microbiota changes, from recent literature, are summarized in Table 2.

## 5. The Tripartite Role in Health and Disease

**Figure 3:**
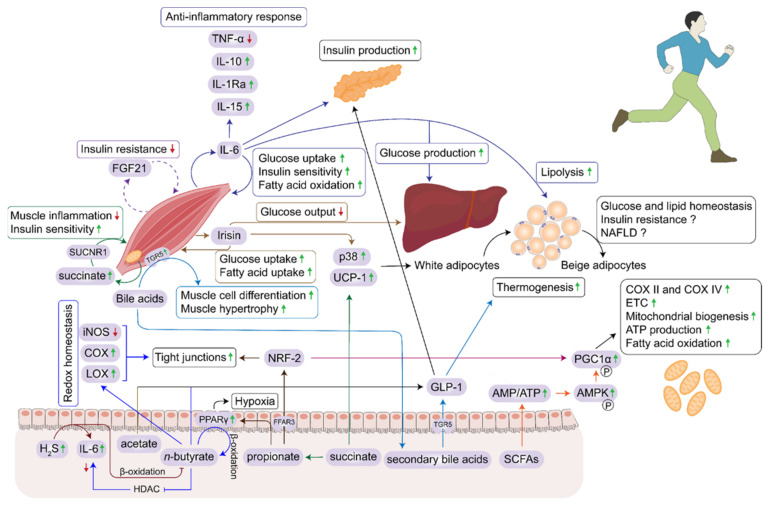
The interface between exerkines and microbiota-derived metabolites for interorgan cross-talk and host energy homeostasis. Thermogenic adipose tissue has been therapeutically targeted for obesity and type 2 diabetes mellitus (T2DM), where microbial secondary bile acids mediated glucagon-like peptide 1 (GLP-1) release via TGR5, enhances thermogenesis. Activation of bile-acid receptor TGR5 in skeletal muscles induces muscle hypertrophy and differentiation. Exercise upregulates TGR5 expression in muscle cells. IL-6 and irisin are exercise induced myokines, however, fibroblast growth factor 21 (FGF21) is a proposed myokine which acts in an autocrine fashion. IL-6 was initially associated with muscle damage, but its advantageous contribution to exercise is elucidated by crosstalk with pancreas, liver and adipose tissue. Butyrate mediated HDAC inhibition leads to downregulation of IL-6. Moreover, butyrate is vital in the assembly of tight junctions in intestinal and vascular endothelial cells by inducing cyclo-oxygenase (COX), lipoxygenase (LOX) and reducing inducible NO synthase (iNOS). Importantly, SCFAs can cross blood brain barrier and regulate the expression of tight junction proteins and rate of transport by decreasing permeability.

The three-way interaction of the gut microbiome, mitochondria, and exercise intervention can coherently induce a greater impact on the re-modelling of the human physiological processes for a balanced and healthy outcome. The appropriate contribution of these factors for human health is evident. Each component has its interdependency on the other, through which they each modulate one another in a dynamic and complex manner. The complex interplay of exerkines and microbiota-derived metabolites is displayed in Figure 3.

The gut microbiome is closely related to various disease states such as T2DM [205]. Several species of gut bacteria are associated with metabolic risk markers of obesity [206] or liver cirrhosis [207]. Metformin, which is the primary therapeutic option for T2DM, has been shown to target gut bacteria [208]. Gut microbiota-derived metabolites such as TMAO have been found to be elevated in circulation and serve as a strong prognostic marker for unfavorable cardiac events and chronic kidney diseases [209,210]. Preeclampsia patients suffer from gut microbiota dysbiosis and increased levels of plasma LPS and TMAO, which are linked to inflammation [211]. Similarly, increased BCAA levels have been associated with obesity and T2DM [20,212]. In IBD, an upregulation of H_2_S production from cysteine by the gut microbiota is observed concomitantly with a downregulation of enzymes implicated in its mucosal detoxification. In colorectal cancer patients, an upregulation of both endogenous and microbial H_2_S production from cysteine is observed at tumor site that might contribute to disease progression [60].

Likewise, mitochondrial functions are enunciated during the pathogenesis and progression of numerous human diseases (cancer, neurodegenerative and cardiovascular disorders, diabetes, etc.) via sequential events of programmed (apoptosis, necroptosis, autophagy, etc.) and unprogrammed (necrosis) cell death [213]. Recently, mitochondria-derived damage-associated molecular patterns (mito-DAMPs) induced inflammatory responses have been linked to liver fibrosis. Mito-DAMPS are released from damaged hepatocytes and directly activate the fibrogenic hepatic stellate cells to drive liver scarring. Dying hepatocytes release mito-DAMPS via efferocytosis, which is controlled by resident liver macrophages and infiltrating myeloid cells (Gr-1+) [214]. Moreover, a study strongly supported the notion that mitochondrial genotype can modulate both host ROS production and the microbial species diversity in the gut, implying that the changes in the gut microbiome associated with common disease phenotypes might be due to the underlying alteration in mitochondrial function, suggesting the possible mechanisms correlating with H_2_O_2_ production and NAD+/NADH redox status [177]. Mt-ROS production can inhibit the activity of PHDs to increase HIF-1α, in hypoxic as well as normoxic conditions [215,216]. On the contrary, hypoxic cancerous cells can reactivate PHDs by increasing α-ketoglutarate, causing a severe metabolic dyshomeostasis, leading to cell death [217].

Studies suggest that mitochondrial signaling can promote epithelial homeostasis if it originates from commensal bacteria and can cause harm if induced by pathogenic bacteria. Microbiome dysbiosis and the resulting disruption of hypoxia emanate in the expansion of facultative anaerobic bacteria in the gut and lead to inflammatory disorders, such as IBD, where depolarized mitochondria tend to accumulate mt-ROS and damage tight and adherens junctions (AJs) [41]. Mt-ROS production in immune cells is induced by SCFAs or invading bacteria via enhanced mitochondrial respiration. During infection and inflammation, the mitochondrial enzyme Irg 1 (Immune-Responsive Gene 1) is activated in macrophages. This enzyme regulates the activity of FAO and enhances OXPHOS and mt-ROS production. Elevated mt-ROS levels are crucial for eradicating invading pathogens by macrophages and neutrophils. The accumulation of mt-ROS in macrophages and neutrophils is mediated by the activation of TLRs 1, 2, or 4 on the plasma membrane or endoplasmic reticulum during bacterial/viral infection. Antimicrobial ROS can directly cause bactericidal effects or activate the NLRP3 inflammasome to produce pro-inflammatory cytokines [218,219,220]. Therefore, certain bacterial pathogens exploit eukaryotic cellular machinery and apoptotic pathways by targeting the mitochondria. *Clostridium difficile* produces mitochondrion-targeting toxins in intestinal epithelial cells to suppress mt-ATP-sensitive potassium channels, which induce mitochondrial membrane hyperpolarization and apoptosis, which in turn subsequently impair epithelial barrier function [221].

Exercise can reduce the risk of many metabolic and non-transferable diseases. Enhanced skeletal muscle activity during exercise promotes microbial diversity by releasing myokines, which suppress systemic inflammation, increase mitochondrial biogenesis, and escalate fatty acid oxidation [164,222], all of which pave the way for a healthy lifestyle. Exercise can also help ameliorate neurodegenerative diseases such as Alzheimer’s disease (AD) by modulating the microbiota composition. The abundance of *Lactobacillus johnsonii* was reported to be positively associated with the β-amyloid content and area in mice. Exercise, along with probiotics, can slow down the detrimental progression of AD, and these benefits can partly be attributed to alterations in the microbiome [223].

NRF-2 activation can be therapeutically targeted for numerous chronic diseases of lung and liver, metabolic disorders, and cancer initiation. Moreover, exercise has a pivotal role in positively regulating the NRF-2 transcription factor which can work as a primary treatment for age-related diseases such as sarcopenia. NRF-2 is a basic leucine zipper protein (encoded by *NFE2L2*) which is widely recognized because of the cytoprotective roles of its target genes, which are about 250, as established by the NRF-2 knockout in mice [224]. These genes encode a plethora of enzymes that regulate reactions of antioxidant metabolism, xenobiotic biotransformation, carbohydrate and lipid metabolism, iron catabolism, protein degradation, and inflammatory pathways [225]. Kelch-like ECH-associated protein 1 (KEAP1) is a E3-ubiquitin ligase substrate adaptor, responsible for the rapid proteasomal degradation of NRF-2, under non-stressful conditions, where it has a half-life of about 15–40 min [226]. Therefore, the functionality of the NRF-2 and KEAP1 axis is important for a range of disease conditions, with underlying oxidative stress and inflammatory mechanisms, such as metabolic, autoimmune disorders and neurological conditions [227]. NRF-2 also regulates mitochondrial function and ROS-dependent skeletal muscle adaptation, in response to exercise intervention, where exercise has shown NRF-2 mediated improvement in the skeletal muscle function of aged mice. NRF-2 promotes Drp1-dependent mitochondrial fission by deubiquitinating and stabilizing Drp1, to diminish mitochondrial disorder in senescent skeletal muscles [228].

## 6. Conclusions

The factors affecting microbiota diversity and composition are multifactorial, and the mechanisms of microbiota-mediated alterations of host cell function, which may occur via the mitochondria or other systems, such as the immune system, are crucial for homeostasis and the prevention of pathogenesis. However, exercise can positively impact the microbial diversity and mitochondrial performance, the underlying mechanisms of which are largely unknown. The identification of personalized effective strategies, including a healthy and active lifestyle and other targeted routines, e.g., a diet plan, will help to nourish the gut microbiota in a way that will reduce the disease burden globally. A major challenge is to limit the toxic metabolite producing microbiota while supporting the growth of bacteria that improve energy metabolism. The use of probiotics, such as *Lactobacillus* and *Bifidobacterium* spp., to modulate the gut microbiota is currently the main strategy for acquiring general health benefits. Understanding of the mechanism of microbial response to exercise, can pave the way for the development of novel therapeutic and nutritional strategies to modulate and customize the microbiota, as well as enhance athletic potential and overall health. The gut microbiota profile can thus be foreseen as a tool to predict potential disorders and the fitness status of an individual.

## Figures and Tables

**Table 2:** Exercise-associated alterations of human gut microbiota.

References	Microbiome Analysis	Exercise and/or Diet	Study Participants and Design	Study Type	Major Outcomes
Bycura et al., 2021 [197]	16S rRNA gene sequencing of V4 region	8-weeks cardiorespiratory exercise (CRE) or resistance training exercise (RTE)	Male (M) and female (F) students, CRE: 28 (21 F, 7 M; aged 18–26), RTE: 28 (17 F, 11 M; aged 18 to 33 years), pre-exercise and post-exercise period (3-weeks each) was used as control	Longitudinal	CRE resulted in a shift in the microbiome composition seen by qualitative distance metrics and Bray-Curtis metric. However, the prompted gut microbiota change did not persist after completion of exercise intervention period. Whereas, RTE did not change the subjects’ gut microbiome.
Craven et al., 2021 [198]	16S rRNA gene sequencing of V3–V4 region	7-weeks training: 3-week normal training (NormTr), 3-week high-volume training (HVolTr, 10, 20 and 30% increase in training volume during each successive week), 1-week taper training (TaperTr, 55% exponential reduction in training volume)	Highly trained middle-distance runners, *n* = 14 (8 M; age 20.7 ± 3.2 years, 6 F; age 22.0 ± 3.4 years), 2 fecal samples collected during NormTr (1 before and 1 after), 1 sample each collected after HVolTr and TaperTr.	Longitudinal	HVolTr decreased *Pasterellaceae, Lachnoclostridium*, *Haemophilus*, *S. parasagunis*, and *H. parainfluenzae* and increased *R. callidu*. TaperTr did not affect the alpha-diversity and global composition of the gut microbiome to pre-HVolTr.
Jaago et al., 2021 [199]	16S rRNA gene sequencing of V3–V4 region	Exercising at high loads during 31-week period in a ‘real-life’ setting, Prebiotic fiber supplement for 30 days, starting at week 27.	18 years old male junior academic rower (BMI 23.3 ± 0.2), Fecal sample collection at week 1, week 27 and week 31.	Longitudinal	Firmicutes/Bacteroidetes ratio increased (as compared to control cohort) while alpha diversity dropped by 20.3%, upon dietary supplementation. Moreover, microbiota shifted from acetate to butyrate producing bacteria, resulting in enhanced abundance of *Prevotella* (41.7%) and *Roseburia* (4.2%), with stable effects on the *Veillonella* spp.
Quiroga et al., 2020 [200]	16S rRNA gene sequencing of V3–V4 region	12-week strength and endurance training exercise	53 children (7 to 12 years old), 39 obese (25 did exercise and 14 were obese control group) and 14 healthy controls.	Longitudinal	Exercise tended to increase *Blautia*, *Dialister* and *Roseburia* genera and counteracted the obesity profile by decreasing Proteobacteria phylum and Gammaproteobacteria class. Exercise significantly inhibited NLRP3 signaling pathway and reduced plasma glucose levels.
Galle et al., 2020 [201]	16S rRNA gene sequencing of V1–V2 region	Physical activity (PA): mean PA level of 3006.2 ± 2973.6 metabolic equivalent (MET)-minutes/week (148–21,090), Mediterranean diet (MD)	140 university students (48.6% males, mean age 22.5 ± 2.9, mean BMI: 22.4 ± 2.8 kg/m^2^)	Cross-sectional	*Streptococcus* and *Dorea* were highly abundant in overweight individuals, while *Ruminococcus* and *Oscillospira* were abundant in participants with lower adherence to MD, and *Lachnobacterium* in subjects with low levels of PA.
Liu et al., 2020 [202]	Shotgun metagenomic sequencing	12-week high-intensity exercise (combination of aerobic and strength training)	39 prediabetic men (age: 31.45–46.56 years, BMI > 23 kg/m^2^)	Longitudinal	No significant difference was observed in alpha or beta diversity between the microbiome before and after exercise. However, compositional analysis showed altered relative abundances of 6 species, belonging to Firmicutes, Bacteroidetes, and Proteobacteria, after exercise, which were correlated with improvements in glucose homeostasis and insulin sensitivity.
Motiani et al., 2020 [203]	16S rRNA gene sequencing of V3–V4 region	Sprint interval (SIT) training: 30 s exercise bouts (4–6) of all out cycling efforts. Moderate-intensity continuous training (MICT): 40–60 min (60% of VO_2_ peak intensity) cycling. Both trainings were carried out six times over 2 weeks span.	26 insulin resistant sedentary subjects (M: 16, F: 10, age: 49 ± 4 year, BMI: 30.5 ± 3 kg/m^2^)	Longitudinal	Exercise training modified the microbiota by increasing Bacteroidetes and decreasing Firmicutes/Bacteroidetes ratio. Moreover, there was a decrease in *Clostridium* and *Blautia* genera. Additionally, VO_2_ peak improved only after SIT. However, both training modes reduced systematic and intestinal inflammatory markers. And colonic glucose uptake was positively associated with Bacteroidetes and inversely related with Firmicutes phylum.
Rettedal et al., 2020 [204]	16S rRNA gene sequencing of V3–V4 region	3 weeks of High-intensity interval training (HIIT) (8–12 × 60 s cycle ergometer bouts)	14 lean (fat mass 21 ± 2%, aged 29 ± 2 years) and 15 overweight (fat mass 33 ± 2%, aged 31 ± 2 years) men	Longitudinal	HIIT significantly increased the aerobic fitness of both groups and improved markers of insulin sensitivity. However, HIIT did not affect the overall bacterial diversity or community structure (α-diversity or β-diversity). However, significant associations were observed between *Coprococcus_3*, *Blautia, Lachnospiraceae_ge* and *Dorea* and insulin sensitivity markers in the overweight group.
